# Investigation of the mechanisms of action behind Electromotive Drug Administration (EMDA)

**DOI:** 10.7717/peerj.2309

**Published:** 2016-08-24

**Authors:** Bor Kos, Juan Luis Vásquez, Damijan Miklavčič, Gregers G.G. Hermann, Julie Gehl

**Affiliations:** 1 Faculty of Electrical Engineering, University of Ljubljana, Ljubljana, Slovenia; 2 Department of Oncology, Copenhagen University Hospital Herlev, Copenhagen, Denmark; 3 Department of Urology, Copenhagen University Hospital Frederiksberg, Copenhagen, Denmark

**Keywords:** Bladder cancer, Mitomycin C, Electromotive Drug Administration, Electroporation

## Abstract

**Objective:**

Bladder cancer is a cause of considerable morbidity worldwide. Electromotive Drug Administration is a method that combines intravesical chemotherapy with local electric field application. Electroporation has been suggested among other mechanisms as having a possible role in the therapy, so the goal of the present study was to investigate the electric fields present in the bladder wall during the treatment to determine which mechanisms might be involved.

**Material and Methods:**

Electromotive Drug Administration involves applying intravesical mitomycin C with direct current of 20 mA delivered through a catheter electrode for 30 min. For numerical electric field computation we built a 3-D nonhomogeneous patient specific model based on CT images and used finite element method simulations to determine the electric fields in the whole body.

**Results:**

Results indicate that highest electric field in the bladder wall was 37.7 V/m. The mean electric field magnitude in the bladder wall was 3.03 V/m. The mean magnitude of the current density in the bladder wall was 0.61 A/m^2^.

**Conclusions:**

The present study shows that electroporation is not the mechanism of action in EMDA. A more likely explanation of the mechanism of action is iontophoretic forces increasing the mitomycin C concentration in the bladder wall.

## Introduction

Bladder cancer is a cause of considerable morbidity and mortality worldwide. The intensive care and treatment of bladder cancer represents a considerable burden to patients and society (Botteman et al., 2003). Intravesical therapy has been used to maximize the exposure of bladder tumors to the drug, while limiting systemic effects and toxicity. Mitomycin C (MMC) is the most widely used drug for intravesical irrigation in low and intermediate risk tumors (Bouffioux et al., 1995; Sylvester, Oosterlinck & Van der Meijden, 2004), while Bacillus Calmette-Guérin (BCG) is recommended for high-risk tumors (Sylvester, Van der Meijden & Lamm, 2002; Shelley et al., 2004; Böhle & Bock, 2004). Intravesical chemotherapy with MMC is used immediately after transurethral resection of tumor (TURBT) to treat circulating tumor cells that can reimplant at other sites of the bladder, or as an ablative effect on residual tumour cells at the resection site (Pan et al., 1989; Brocks, Büttner & Böhle, 2005). MMC is also used in series of 8-weekly intravesical irrigations for patients with high risk of recurrence (Tolley et al., 1996; Sylvester, Oosterlinck & Witjes, 2008).

MMC migrates poorly deeper into the lamina propria, which may explain MMC’s poor efficacy treating T1 tumors invading this layer of the mucosa. Electrokinetic forces may accelerate and increase drug delivery across the urothelium. Electromotive Drug Administration (EMDA) is a method that combines intravesical chemotherapy with local electric field application. Di Stasi et al. (1999) suggested in an *ex vivo* model that concentrations of MMC may be increased in all the layers of the bladder wall, when the drug was delivered with EMDA. Clinical studies where EMDA/MMC was applied showed lower recurrence rates than MMC alone, and equivalent efficacy for the treatment of high-risk bladder tumors when compared to BCG (Di Stasi et al., 2003; Di Stasi et al., 2006) with few side effects noted. Thus EMDA method seems safe and effective.

It has been proposed that EMDA leads to the combination of several electro-molecular interactions; iontophoresis, electroosmosis/electrophoresis, and electroporation (Di Stasi et al., 1997). All these phenomena may potentially be responsible for electromotive transport of drug molecules through biological membranes and into the underlying tissue when the electric field is applied. EMDA method is being used experimentally in relatively few centers, possibly due to the fact that the mechanisms of action are poorly understood. However, the actual worldwide shortage of BCG has led to the use of alternative methods and increased use of EMDA/MMC. Consequently there is a need to further elucidate the mechanisms of action of this treatment, which led to the present study.

The objective of this study is to perform calculations of the electric field present in the bladder wall during EMDA treatments, and in that way estimate what biological mechanisms may be at play when the tissue is exposed to this particular electric field.

## Materials and Methods

The main goal of the following steps was to build an *in-silico* representation of a human body during the delivery of an EMDA treatment. This was done by reconstructing the anatomical shape of the body and the relevant tissues from CT images and then using a computational approach to determine the relevant electric quantities in the tissues: electric field and current density. These quantities were then analyzed to get an insight into the possible mechanisms of EMDA.

### Delivery setup

EMDA is administered by a battery powered current generator (Physionizer^®^) that delivers a controlled electric current of 0–30 mA/0–55 V DC, which is passed between electrodes: (1) the active intravesical electrode is a silver spiral integrated to the tip of a specifically designed transurethral catheter, (2) two dispersive ground electrodes, placed in the lower abdominal skin. The operator sets the active electrode’s polarity and current intensity on the generator.

**Figure 1:**
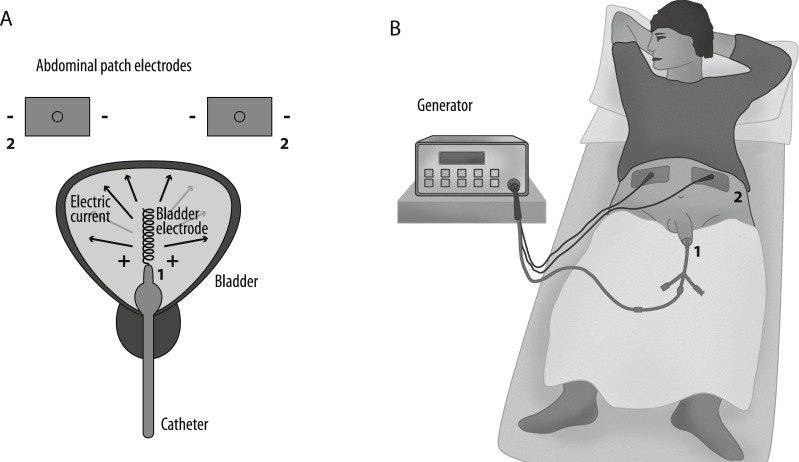
Schematic illustration of the EMDA application system. (A) Numbers indicate the electrodes: 1, helical bladder catheter electrode; 2, abdominal patch electrodes. (B) The catheter is inserted in the urinary bladder and connected to the current generator.

When applying intravesical EMDA with MMC for non-muscle invasive bladder cancer, the catheter is inserted into the bladder followed by instillation of 40 mg of MMC in 50 ml 0.9% NaCl solution. The catheter and the ground electrodes are connected to the generator, and the active electrode is set to positive polarity. The generator gradually increases the current to a predetermined level (20–25 mA) at a rise rate of 50 mA/s. The total treatment time is 30 min. This setup is shown in Fig. 1.

### Segmentation of medical images

Different tissues have different electrical properties. To be able to accurately compute the electrical quantities in the body we need to differentiate between these tissues and assign them their appropriate properties. Abdominal CT images of a patient treated with EMDA were segmented into the following tissues (bladder wall, bladder lumen, small intestine, prostate, muscle, bone, fat, and internal air) using ITK-Snap version 2.4 (Yushkevich et al., 2006). The patient was a participant in a clinical trial where EMDA was tested, and the Ethics Committee of the Capital Region of Denmark approved this trial (study number H-1-2012-050). As the current study is not part of the clinical trial, permission for the use of the CT images was obtained from the patient orally.

A radiologist verified the segmentation. The pixel size in the segmentation was 0.781 mm, while the slice separation was 3 mm. In total 72 slices were segmented, corresponding to 14 slices below the bladder and 3 slices above the top edge of the external electrodes.

### 3-D model building

**Figure 2:**
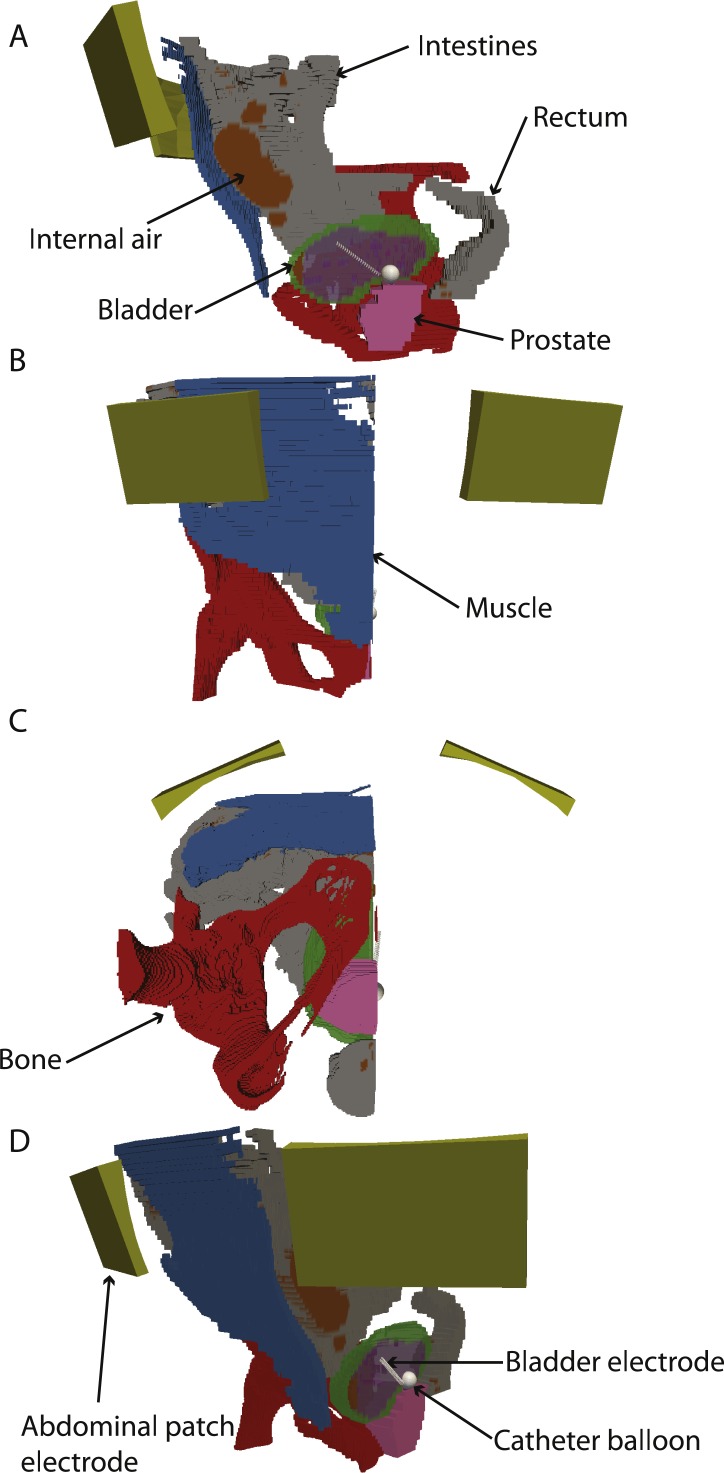
3-D model with electrodes and different tissues. (A–C) sagittal, coronal and axial views. (D) rotated 3D view. The abdominal and bladder electrodes are indicated. In all panels, the bladder, intestine, prostate and internal air are shown, but clipped to only half of the model for clarity.

A 3-D model of the patient’s anatomy was constructed in COMSOL Multiphysics version 5.2 (Comsol AB, Stockholm, Sweden). The outside of the body was constructed using a planar contour method using an algorithm written in Matlab (Mathworks, Natick, MA, USA) (Sel, Lebar & Miklavcic, 2007). The tissues of the interior of the body were taken into account by building a spatial conductivity matrix in Matlab by assigning conductivity values based on the segmentation (Aström et al., 2009). The tissue properties were taken from the literature (Gabriel, Lau & Gabriel, 1996a; Gabriel, Lau & Gabriel, 1996b) and the actual values used are reported in Table 1. The conductivity of the saline used for bladder irrigation was also taken from the literature (Stogryn, 1971). Electrodes were added by CAD modelling directly in COMSOL based on the dimensions of the actual electrodes. The balloon of the catheter (12 mm diameter) was placed right above the bladder neck. The catheter electrode consists of a helical wire with a diameter of 0.4 mm and 20 turns. The major radius of the helix was 1.25 mm, while the turn pitch was 1.6 mm; therefore, the total combined length of the electrode is 32 mm. This electrode shape limits the intensity of the electric field and current density around the electrode. The outer electrodes were modelled as a patch with outer dimensions of 5 × 7 cm. The resulting model is shown in Fig. 2. The catheter balloon was modelled as a perfect insulator, so it was excluded from the computation.

**Table 1:** Electrical conductivity values used in the computational model.

Tissue	Conductivity [S/m]	Reference
Bladder wall	0.2	Gabriel, Lau & Gabriel (1996a), Gabriel, Lau & Gabriel (1996b)
Bladder lumen	1.5	Stogryn (1971)
Prostate	0.41	Gabriel, Lau & Gabriel (1996a), Gabriel, Lau & Gabriel (1996b)
Fat	0.012	Gabriel, Lau & Gabriel (1996a), Gabriel, Lau & Gabriel (1996b)
Muscle	0.2	Gabriel, Lau & Gabriel (1996a), Gabriel, Lau & Gabriel (1996b)
Intestines	0.4	Gabriel, Lau & Gabriel (1996a), Gabriel, Lau & Gabriel (1996b)
Bone	0.02	Gabriel, Lau & Gabriel (1996a), Gabriel, Lau & Gabriel (1996b)

### Computational setup

We used the stationary finite element method solvers in COMSOL Multiphysics to solve the Laplace equation for electric potential in the 3-D model of the patient’s anatomy. The model was discretized using a free tetrahedral mesh, which consisted of 2,779,405 elements. We used the conductive media DC interface in COMSOL Multiphysics to run a static simulation of the applied current density and electric fields inside the body. The electrodes were driven with a current-source terminal boundary condition, while the skin was taken into account only at the skin-electrode interface, by using a built-in thin resistive layer boundary condition in COMSOL. The results were extracted on a grid with data points positioned at the centers of the image elements in the original anatomical CT scans.

## Results

### Global quantities

Since the electrodes were driven by a current source (20 mA), it is possible to compute the resulting electrode voltages with the data from the manufacturer’s specifications. The voltage between the two electrodes was 13.1 V, which is within the generator specification and corresponds well to the expected voltage. Unfortunately, the generator does not specify the output voltage at the time of the treatment so the exact value used in the treatment is not available for the validation of the model.

### Applied fields in the body

Electric fields in the whole body were extracted. The highest magnitude of electric field in the simulations was 271 V/m, in the subcutaneous fat tissue near the surface electrodes. The maximum values in the bladder wall were 71.9 V/m. The mean and SD of electric field magnitude in the bladder wall were 6.0 V/m and 7.1 V/m, respectively.

**Figure 3:**
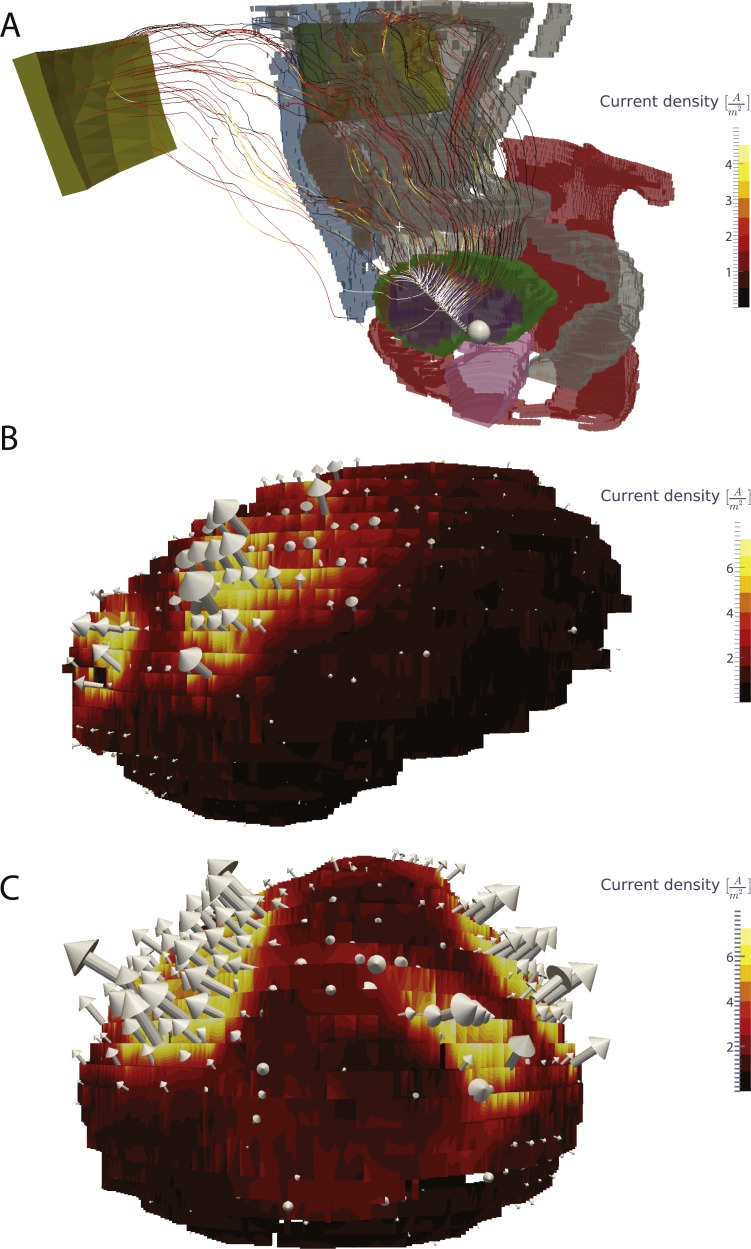
Visualization of the current in the whole body and the current density in the bladder wall. (A) Current lines from the bladder electrodes to the abdominal patch electrodes. (B) Surface plot of the current density on the bladder wall in the sagittal view (from the left side). (C) Surface plot of the current density in a coronal view (from the front). The current density is markedly higher in the upper anterior bladder wall in comparison to the posterior and lower bladder walls.

The largest current density was found around the catheter electrode: 134 A/m^2^. The largest current density in the bladder wall was 14.4 A/m^2^. Mean current density in the bladder wall was 1.2 A/m^2^, with the standard deviation being 1.4 A/m^2^. The highest current density in all other tissues was 35.6 A/m^2^, however this value was found in a single voxel and may be due to numerical error. The current density is mostly perpendicular to the bladder, although the current density is much higher at the anterior part than at the posterior as would be expected based on electrode positioning Fig. 3.

**Figure 4:**
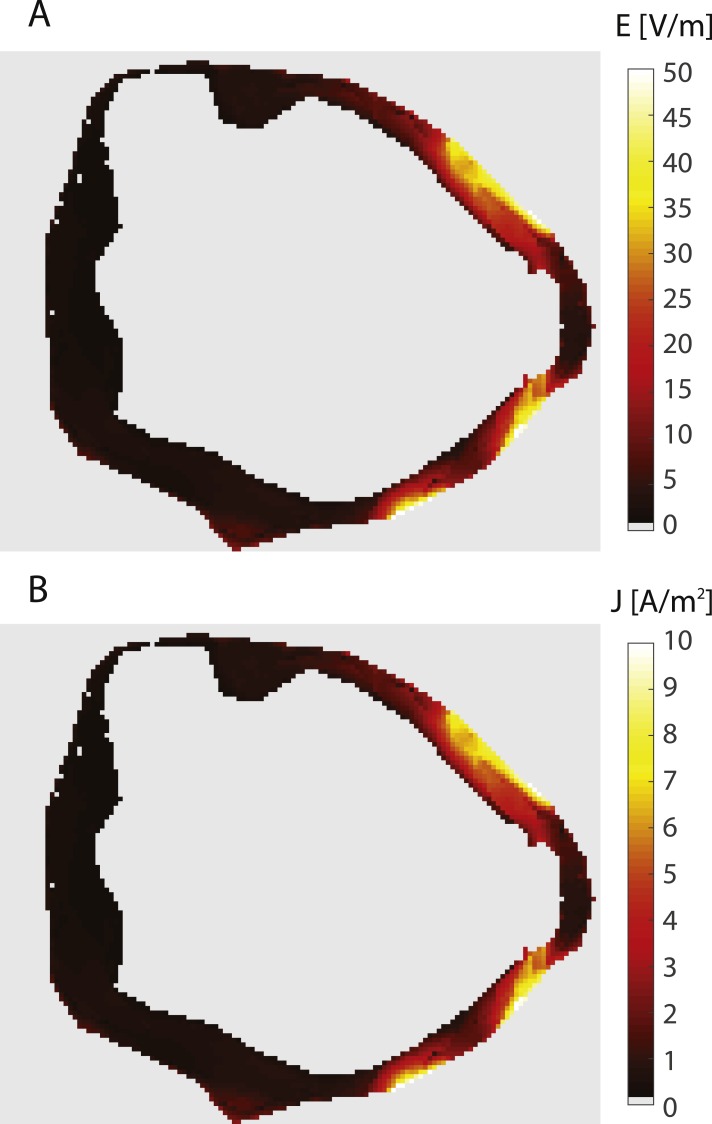
Electric field and current density in the bladder wall. The figure shows an axial slice at the vertical center of the bladder wall. Both panels show the same slice. The panels show: (A) Electric field, (B) Current density.

## Discussion

Based on the values of electric fields, we can conclude that electroporation is highly unlikely to be the predominant mechanism of action in EMDA. Typically, electroporation occurs at electric field strengths that are above 40,000 V/m (Cemazar et al., 1998; Miklavcic et al., 1998; Gehl et al., 1999; Pucihar et al., 2011) also on urothelial cells (JL Vásquez, 2013, unpublished data), while the electric fields reached established by EMDA are well below even 100 V/m. Although electroporation thresholds decrease with increasing duration of electric field application, the lowest reported in the literature is above 10,000 V/m for 100 ms pulse duration (Pucihar et al., 2011). The highest amplitude of the induced transmembrane voltage (ITV) according to Schwann’s equation (Schwan, 1957) is determined by the equation ITV_Max_ = 1.5*ER*, where *E* is the electric field strength to which the cell of radius *R* is exposed. Based on the size of the cells used by Pucihar et al. in their experiments, the highest transmembrane voltage at 100 ms pulses was approximately 150 mV. The superficial layer of the bladder contains polyhedral cells of up to 250 µm in diameter (Khandelwal, Abraham & Apodaca, 2009). Given the value of the electric field in the bladder, the highest expected ITV during the treatment is 14 mV or lower, which is well within physiological values of transmembrane potential. The other layers of the bladder contain much smaller cells, which would result in even lower ITV.

Typically, iontophoresis setups for trans-dermal drug delivery use current densities in the range of 0.1–0.5 mA/cm^2^ (Kalia et al., 2004), which is equal to 1–5 A/m^2^ and therefore very close to the values produced by our model. The values of current density in the bladder wall support the hypothesis, that iontophoresis is involved in EMDA, and that is likely to be the contributing factor for reducing recurrence, due to the increase of the drug concentration near the point of excision.

Due to the placement of the abdominal electrodes on the front part of the abdominal wall, the predominant direction of the current flow is towards the front, as can be seen in Figs. 3 and 4. This means that the current density of the posterior bladder wall could be below the current density required for efficient iontophoretic flow, i.e., the treatment would be more successful for tumors located on the front half of the bladder than for the back half.

The present study is limited by the fact that it is based on a single patient, and that the exact positioning of the intravesical electrode during the treatment is unknown. Additionally, the variability between patients would affect the values of electrical quantities in the tissue. However, even with these variations, the difference in the electric fields and current density would not be so great to change our findings.

Our study shows that electroporation is *not* the mechanism of action in EMDA. However, electroporation combined with chemotherapy (electrochemotherapy) is a novel and interesting treatment modality in use for the treatment of cutaneous tumors and in clinical trials for tumors in internal organs (Campana et al., 2009; Matthiessen et al., 2011; Edhemovic et al., 2011; Miklavčič et al., 2014). Also for bladder cancer, there are interesting perspectives for electrochemotherapy, both using MMC and cisplatin (Vásquez, Gehl & Hermann, 2012; Vásquez et al., 2015). These data on cisplatin used in electrochemotherapy experiments may give inspiration for new advances in iontophoretic drug delivery.

## Conclusion

Based on numerical computational modelling based on realistically dimensioned models of EMDA treatment, we suggest that iontophoretic forces are predominant in this treatment. Electrode placements could mean that the anterior wall of the bladder receives a higher treatment field than other parts of the bladder. Also, due to the presence of a small air pocket in the bladder, the location directly above the air pocket receives a reduced treatment. Based on the numerical results alternate electrode positions should be considered if the original location of the excised tumor is not on the anterior bladder wall.

## Additional Information and Declarations

### Data Availability

The following information was supplied regarding data availability: Kos, Bor; Luis Vásquez, Juan; Miklavcic, Damijan; G Hermann, Gregers; Gehl, Julie (2016): Investigation of the mechanisms of action behind Electromotive Drug Administration (EMDA). Figshare https://dx.doi.org/10.6084/m9.figshare.3118759.v1.
